# Choroidal neovascularization in angioid streaks following microincision vitrectomy surgery: a case report

**DOI:** 10.1186/1471-2415-13-29

**Published:** 2013-07-05

**Authors:** Satoshi Katagiri, Takaaki Hayashi, Hirotsugu Takashina, Katsuya Mitooka, Hiroshi Tsuneoka

**Affiliations:** 1 Department of Ophthalmology, The Jikei University School of Medicine, 3-25-8 Nishi-shimbashi, Minato-ku, Tokyo 105-8461, Japan; 2 Department of Ophthalmology, Daisan Hospital, The Jikei University School of Medicine, Tokyo, Japan

**Keywords:** Angioid streaks, Choroidal neovascularization, Vitrectomy surgery, Anti-vascular endothelial growth factor treatment, Photodynamic therapy, Pseudoxanthoma elasticum

## Abstract

**Background:**

Patients with angioid streaks are prone to developing subretinal hemorrhage after ocular or head injury due to the brittleness of Bruch’s membrane. However, there have been no reports of any angioid streak patients in whom choroidal neovascularization occurred after vitrectomy surgery. We report herein a patient with angioid streaks who developed choroidal neovascularization after vitrectomy surgery for epiretinal membrane.

**Case presentation:**

A 76-year-old man presented with distorted vision in his left eye, with a best corrected visual acuity of 1.2 and 0.6 in his right and left eyes, respectively. Fundus examination showed angioid streaks in both eyes and epiretinal membrane only in the left eye. The patient underwent 23-gauge three-port pars plana vitrectomy with removal of the epiretinal membrane combined with cataract surgery. Internal limiting membrane in addition to the epiretinal membrane were successfully peeled and removed, with indocyanine green dye used to visualize the internal limiting membrane. His left best corrected visual acuity improved to 0.8. An elevated lesion with retinal hemorrhage due to probable choroidal neovascularization was found between the fovea and the optic disc in the left eye at 7 weeks after surgery. Since best corrected visual acuity decreased to 0.15 and the hemorrhage expanded, posterior sub-Tenon injection of triamcinolone acetonide was performed. However, no improvement was observed. Even though intravitreal bevacizumab injection was performed a total of five times, his best corrected visual acuity remained at 0.1. Subsequently, we performed a combination treatment of a standard-fluence photodynamic therapy and intravitreal ranibizumab injection, with additional intravitreal ranibizumab injections performed 3 times after this combination treatment. Best corrected visual acuity improved to 0.5 and the size of the choroidal neovascularization markedly regressed at 4 months after the combined treatment.

**Conclusion:**

Development of choroidal neovascularization could possibly occur in elderly patients with angioid streaks after vitrectomy surgery. In such cases, a combination of photodynamic therapy and intravitreal ranibizumab injection may be considered for initial treatment of the choroidal neovascularization.

## Background

Angioid streaks (AS) are visible irregular crack-like dehiscences radiating from the optic nerve to the peripheral retina. Approximately 70% of AS patients have pseudoxanthoma elasticum (PXE) characterized by changes in the elastic tissue of the skin [1]. Patients with AS are usually asymptomatic unless the lesions extend towards the fovea or develop complications such as traumatic Bruch’s membrane rupture or choroidal neovascularization (CNV). Several studies on the relationship between ocular trauma and subretinal hemorrhage in patients with AS have been reported [2,3]. There is also a report of an AS patient who developed CNV after indirect trauma to the eye itself [4]. It has also been reported that 15% of AS patients who suffer head injuries develop significant visual impairment [5]. However, to our knowledge, there have been no reports of any AS patients in whom CNV occurred after vitrectomy surgery.

We report herein a case of an AS patient in whom CNV occurred at 7 weeks after microincision vitrectomy surgery for removal of epiretinal membrane (ERM).

## Case presentation

**Figure 1:**
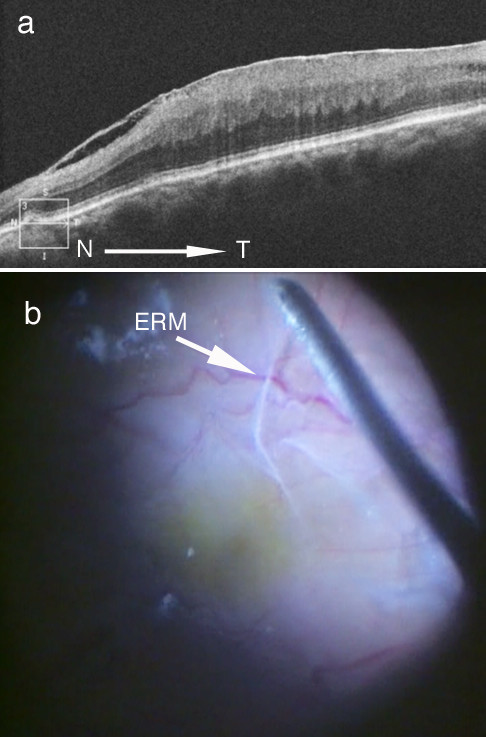
**Optical coherence tomography before surgery and fundus photograph of patient during vitrectomy surgery. a**) Epiretinal membrane (ERM) in optic coherence tomography before vitrectomy surgery. **b**) Fundus photograph during vitrectomy surgery. The ERM is successfully peeled before ILM peeling.

A 76-year-old male patient with a medical history of hypertension but no eye surgery reported distorted vision in the left eye. Best corrected visual acuity (BCVA) at his initial examination was 1.2 (with +1.75 diopter (dpt), cylinder (cyl) -1.25 dpt Ax 110°) in his right eye and 0.6 (with +2.75 dpt, cyl. -1.50 dpt Ax 90°) in his left eye. Using the IOLMaster 500 (Carl Zeiss Meditec AG, Dublin, CA, USA), his axial lengths were determined to be 23.3 and 23.1 mm in his right and left eyes, respectively. Except for mild senile cataracts in the anterior segments and media of both of his eyes, there were no abnormalities found. Intraocular pressures were 13 mmHg on the right and 11 mmHg on the left. Fundus examination showed ERM in the left eye, in addition to bilateral AS. Although spectral-domain optical coherence tomography (OCT; Cirrus HD-OCT, Carl Zeiss Meditec AG) using the high-definition 5-line raster scan protocol (horizontal scan of 6 mm) revealed ERM, CNV was not observed in the macula of his left eye (Figure 1a). The patient subsequently underwent 23-gauge three-port pars plana vitrectomy with removal of ERM combined with cataract surgery at The Jikei University, Daisan Hospital. Indocyanine green dye was used to visualize the internal limiting membrane (ILM). The ILM in addition to the ERM were successfully peeled and removed (Figure 1b). No retinal hemorrhage was noted during the vitreous surgery (Figure 1b), and his left BCVA subsequently improved to 0.8.

**Figure 2:**
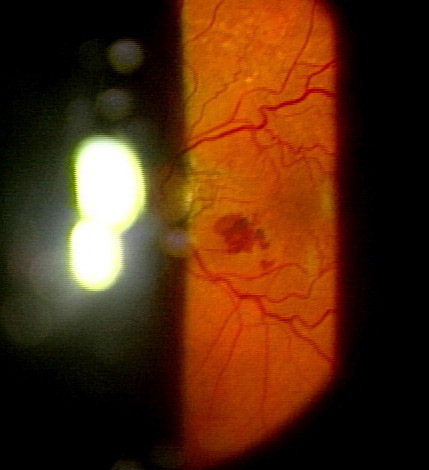
**Left fundus finding 7 weeks after the vitrectomy surgery.** Photograph shows an elevated lesion with retinal hemorrhage that is most likely due to probable choroidal neovascularization found between the fovea and the optic disc.

Seven weeks after the vitrectomy surgery, an elevated lesion with retinal hemorrhage due to probable CNV was found between the fovea and the optic disc in his left eye (Figure 2), although he had no history of any ocular trauma after the surgery. One week later, his left BCVA decreased to 0.15 and the retinal hemorrhage expanded. Although posterior sub-Tenon injection of triamcinolone acetonide was performed, no visual improvement was seen.

**Figure 3:**
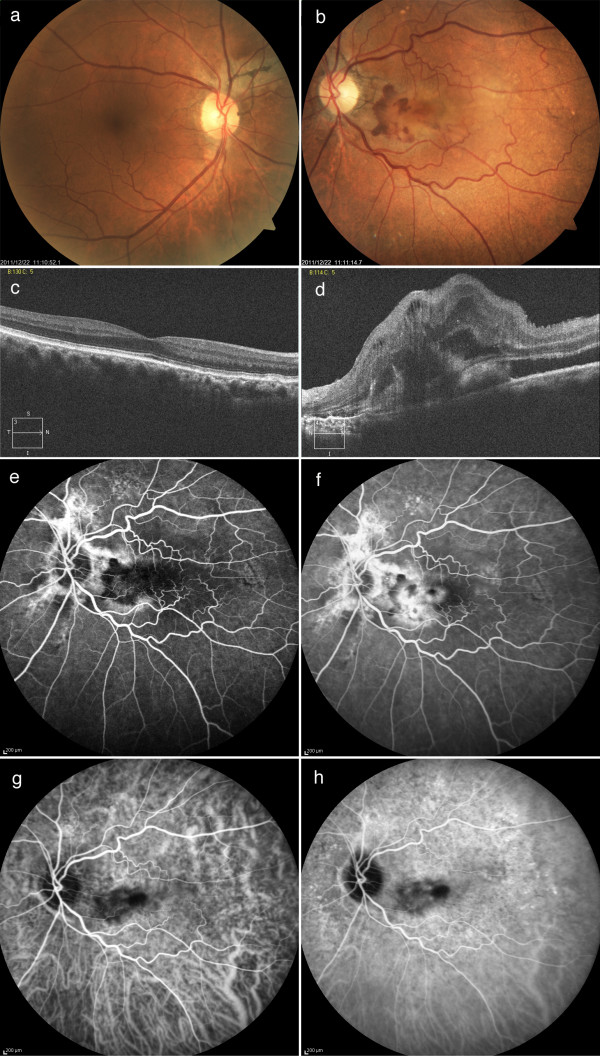
**Fundus findings prior to the initial anti-vascular endothelial growth factor therapy. a**, **b**) Fundus photographs show angioid streaks in the right (**a**) and left (**b**) eyes and choroidal neovascularization (CNV) in the left macula (**b**). **c**, **d**) Optical coherence tomography shows no abnormal finding in the right eye (**c**) and Gass type 2 CNV with macular edema in the left eye (**d**). **e**, **f**) Fluorescein angiograms show well-defined (classic) CNV in the early (**e**) to late phases (**f**) in the left macula. **g**, **h**) Indocyanine green angiograms show CNV is visualized from the early (**g**) to late phases (**h**) in the left macula.

At 12 weeks after the vitrectomy surgery at The Jikei University Hospital, his BCVA was 0.08 in his left eye. Fundus examination revealed a radiating dark brownish line from the optic disc head in his right (Figure 3a) and left (Figure 3b) eyes, and there was an elevated subretinal lesion with retinal hemorrhage in his left macula (Figure 3b). Although OCT showed no abnormal findings in his right eye (Figure 3c), Gass type 2 CNV with macular edema was observed in his left eye (Figure 3d). Fluorescein angiography (FA) revealed well-defined (classic) CNV from the early (Figure 3e) to late phases (Figure 3f) in the left macula. Using indocyanine green angiography (ICGA), we were able to visualize the CNV from the early (Figure 3g) to late phases (Figure 3h).

**Figure 4:**
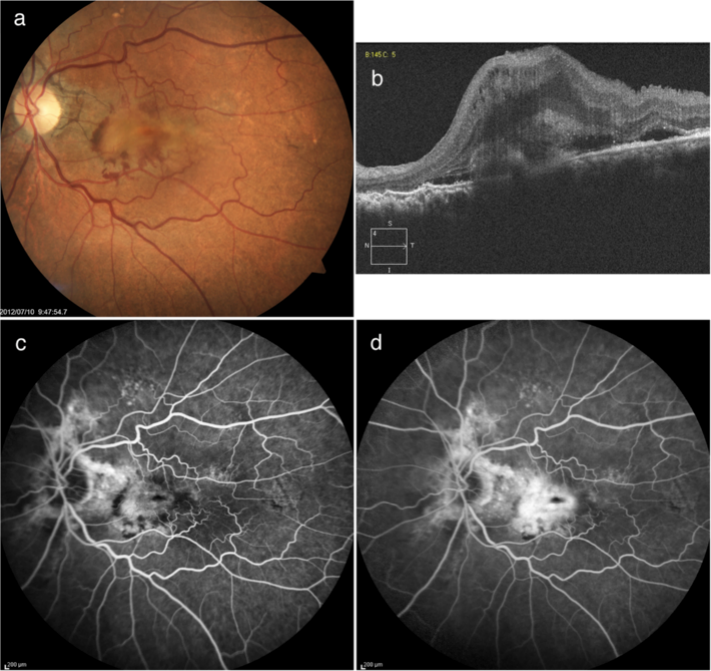
**Left fundus findings after 5 intravitreal bevacizumab injections. a**) Fundus photograph indicates there is no regression of the choroidal neovascularization (CNV). **b**) Optical coherence tomography also confirms there is no regression of the CNV. **c**, **d**) Fluorescein angiograms show evidence of classic CNV in the early (**c**) to the late phases (**d**).

Over the next 6 months, a total of 5-time intravitreal injections of the anti-vascular endothelial growth factor (VEGF) drug, bevacizumab (Avastin, Genentech, San Francisco, CA, USA), were given. His left BCVA remained at 0.1. Fundus examination demonstrated that there was no regression of the CNV (Figure 4a), as was confirmed by OCT (Figure 4b). The FA images indicated there was a foveal lesion with evidence of classic CNV in the early phase (Figure 4c) and an extended high fluorescent lesion in the late phase (Figure 4d).

**Figure 5:**
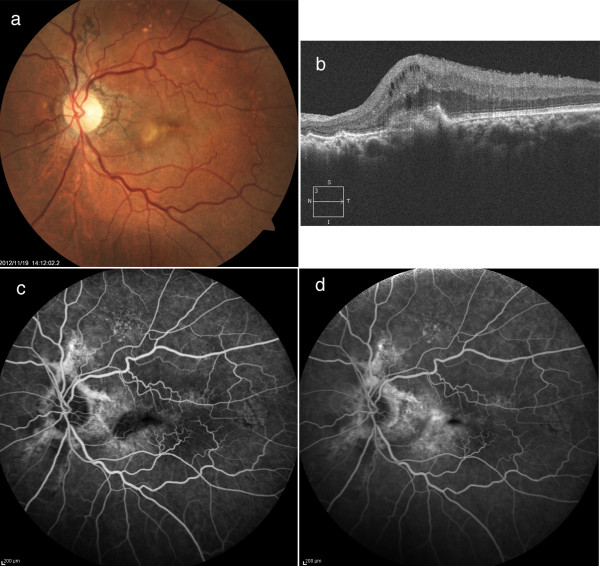
**Left fundus findings 4 months after the combination treatment. a**) Fundus photograph shows marked regression of the choroidal neovascularization (CNV). **b**) Optical coherence tomography shows significant but incomplete regression of CNV, and persistence of intra-retinal cysts. **c**, **d**) Fluorescein angiograms show that leakage from the CNV almost completely disappears between the early (**c**) and late phases (**d**).

Subsequently, we performed standard-fluence photodynamic therapy (PDT: laser fluence set at 50 J/cm^2^) with full-dose verteporfin (Visudyne; Novartis Pharma AG, Basel, Switzerland) in combination with an intravitreal ranibizumab (Lucentis, Genentech) (IVR) injection. After this combination treatment, 3 additional IVR treatments were performed. At 4 months after the combination treatment, his BCVA improved to 0.5. There was a marked regression of the CNV size observed in both the fundus (Figure 5a). OCT showed significant but incomplete regression of CNV, and persistence of intra-retinal cysts (Figure 5b). The FA images revealed that leakage from the CNV noted in the early phases (Figure 5c) almost completely disappeared by the late phases (Figure 5d).

**Figure 6:**
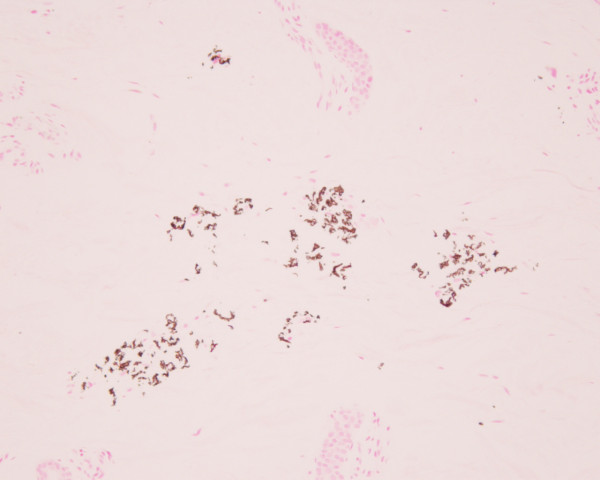
**Histological findings of a skin biopsy on the neck.** The histological section reveals short and broken elastic fibers with dark staining of calcium deposits by Von Kossa staining in the reticular dermis, convincing a diagnosis of pseudoxanthoma elasticum.

Examination of the skin on the neck showed suggestive of PXE. A skin biopsy was performed from the lesion on the neck. The histological section of the biopsy revealed short and broken elastic fibers with dark staining of calcium deposits by Von Kossa staining in the reticular dermis (Figure 6), convincing a diagnosis of PXE.

## Discussion

In this report, we describe a single AS patient in whom CNV developed 7 weeks after vitrectomy surgery for ERM. To date, there have been no other reports of any AS patients who have developed CNV after vitrectomy surgery.

Numerous studies have reported the incidence of CNV to vary between 72%–86% in AS patients [5]. It is reported that the risk of developing CNV increases with age [6]. Other risk factors comprise the width, length and location of the AS. Several studies have reported that wider and longer AS are associated with higher risk of CNV, and that the risk of CNV development is especially higher if AS are located within one optic disc diameter from the foveola [5,7,8].

As an external factor, ocular trauma can be a risk factor for the development of subretinal hemorrhage [2,3]. In fact, AS patients are likely to develop breaks of Bruch’s membrane even after relatively mild ocular head injuries, since their Bruch’s membrane is brittle [5]. In addition, it has also been reported that an AS patient developed CNV 4 months after an indirect ocular trauma [4]. Thus, it is possible that CNV can occur due to either direct or indirect ocular trauma.

Regarding the relationship between vitrectomy surgery and CNV development, there have been some studies that have reported finding CNV after vitrectomy surgery for idiopathic macular hole [9-12]. Although the pathogenesis underlying the development of CNV after macular hole surgery has yet to be completely clarified, it has been suggested that the most plausible explanation is an age-related degenerative change in the retinal pigment epithelium (RPE) and Bruch’s membrane. This is supported by the fact that there is a higher prevalence of CNV development after macular hole surgery in older patients [10,13]. In addition, as has been reported in a previous study, mechanical trauma during the ILM peeling in the macular hole surgery might have played a role in the pathogenesis of the CNV that subsequently developed in the patient [10]. In fact, this is similar to the current case, as our patient had brittleness of Bruch’s membrane and RPE because of AS and aging and thus, underwent vitrectomy surgery with ERM and ILM peeling, after which he developed CNV.

When treating CNV secondary to AS, previous studies have described the use of monotherapy treatments employing PDT or anti-VEGF therapies. For example, Arias et al. reported that PDT was not effective in the treatment of CNV in the macula [14], while Browning et al. showed that PDT delayed, but did not permanently prevent visual loss associated with the CNV in AS [15]. However, in other cases it has been reported that injection of intravitreal bevacizumab (IVB) was effective as a long-term control tool for CNV associated with AS [16,17]. Moreover, it has been reported that IVR was also effective in treating CNV associated with AS [18-20]. Even so, after the patient in the current case received 5-time IVB injections, the CNV remained active. This suggested that there was a decrease in the biologic effect after repeated intravitreal anti-VEGF injections of the same drug, such as bevacizumab [21-23]. An *in vitro* experiment demonstrated that the absence or presence of hyaluronan, which is a major component in the vitreous body, may be associated with the clinical efficacy of IVB because of the higher affinity of hyaluronan for bevacizumab as compared to that for ranibizumab [24]. Christoforidis et al. revealed that clearance rates for intravitreally placed bevacizumab or ranibizumab in vitrectomized or lensectomized rabbit model eyes are faster than those in control eyes [25]. Based on these previous findings in conjunction with the bevacizumab tachyphylaxis and the vitrectomized-pseudophakic condition that was present, we decided to use ranibizumab instead of bevacizumab in all of the subsequent treatments for this patient. Recently, it has been reported that the combination of IVR and reduced-fluence PDT for CNV associated with AS was effective in the regression of CNV and in improving (or stabilizing) the visual acuity [26,27]. Based on the above-mentioned findings, we performed the combined therapy of full-dose PDT and IVR instead of IVR monotherapy. Our current results support the effectiveness of the combined therapy, as the combination therapy of full-dose PDT and IVR was effective, with improvement of the visual acuity and regression of the CNV ultimately seen in our patient.

## Conclusions

Development of CNV can occur in elderly patients with AS after vitrectomy surgery. In such cases, the combination of PDT and anti-VEGF therapies may be considered for use as the initial treatment of CNV.

### Consent

Written informed consent was obtained from the patient for publication of this case report and any accompanying images. A copy of the written consent is available for review by the Editor of this journal.

## Competing interests

The authors declare that they have no competing interests.

## Authors’ contributions

SK drafted the manuscript and reviewed the literature. TH examined and managed the patient and critically analyzed the manuscript. HT examined and managed the patient. KM offered valuable insight into the treatment of the patient. HT offered valuable insight into the treatment of the patient and critically analyzed the manuscript. All authors read and approved the final manuscript.

## Pre-publication history

The pre-publication history for this paper can be accessed here:

http://www.biomedcentral.com/1471-2415/13/29/prepub

## References

[B1] Connor PJ Jr Juergens JL Perry HO Hollenhorst RW Edwards JE Pseudoxanthoma elasticum and angioid streaks. A review of 106 cases Am J Med 1961 30 537 543 10.1016/0002-9343(61)90078-X 13695083

[B2] Britten MJ Unusual traumatic retinal haemorrhages associated with angioid streaks Br J Ophthalmol 1966 50 540 542 10.1136/bjo.50.9.540 5919263 PMC506266

[B3] Levin DB Bell DK Traumatic retinal hemorrhages with angioid streaks Arch Ophthalmol 1977 95 1072 1073 10.1001/archopht.1977.04450060159017 869751

[B4] Pandolfo A Verrastro G Piccolino FC Retinal hemorrhages following indirect ocular trauma in a patient with angioid streaks Retina 2002 22 830 831 10.1097/00006982-200212000-00035 12476123

[B5] Georgalas I Papaconstantinou D Koutsandrea C Kalantzis G Karagiannis D Georgopoulos G Ladas I Angioid streaks, clinical course, complications, and current therapeutic management Ther Clin Risk Manag 2009 5 81 89 19436620 PMC2697526

[B6] Shilling JS Blach RK Prognosis and therapy of angioid streaks Trans Ophthalmol Soc U K 1975 95 301 306 1064229

[B7] Mansour AM Ansari NH Shields JA Annesley WH Jr Cronin CM Stock EL Evolution of angioid streaks Ophthalmologica 1993 207 57 61 10.1159/000310407 8272342

[B8] Mansour AM Shields JA Annesley WH el-Baba F Jr Tasman W Tomer TL Macular degeneration in angioid streaks Ophthalmologica 1988 197 36 41 10.1159/000309915 2460816

[B9] Natarajan S Mehta HB Mahapatra SK Sharma S A rare case of choroidal neovascularization following macular hole surgery Graefes Arch Clin Exp Ophthalmol 2006 244 271 273 10.1007/s00417-005-0004-9 16044324

[B10] Oh HN Lee JE Kim HW Yang JW Yun IH Occult choroidal neovascularization after successful macular hole surgery treated with ranibizumab Clin Ophthalmol 2012 6 1287 1291 22927741 10.2147/OPTH.S33650 PMC3422150

[B11] Tabandeh H Smiddy WE Choroidal neovascularization following macular hole surgery Retina 1999 19 414 417 10.1097/00006982-199909000-00008 10546937

[B12] Berinstein DM Hassan TS Williams GA Margherio RR Ruby AJ Garretson BR Surgical repair of full-thickness idiopathic macular holes associated with significant macular drusen Ophthalmology 2000 107 2233 2239 10.1016/S0161-6420(00)00417-6 11097602

[B13] Tabandeh H Smiddy WE Sullivan PM Monshizadeh R Rafiei N Cheng L Freeman W Characteristics and outcomes of choroidal neovascularization occurring after macular hole surgery Retina 2004 24 714 720 10.1097/00006982-200410000-00005 15492624

[B14] Arias L Pujol O Rubio M Caminal J Long-term results of photodynamic therapy for the treatment of choroidal neovascularization secondary to angioid streaks Graefes Arch Clin Exp Ophthalmol 2006 244 753 757 10.1007/s00417-005-0131-3 16228218

[B15] Browning AC Chung AK Ghanchi F Harding SP Musadiq M Talks SJ Yang YC Amoaku WM United Kingdom PDTUG Verteporfin photodynamic therapy of choroidal neovascularization in angioid streaks: one-year results of a prospective case series Ophthalmology 2005 112 1227 1231 10.1016/j.ophtha.2005.02.011 15921757

[B16] Teixeira A Moraes N Farah ME Bonomo PP Choroidal neovascularization treated with intravitreal injection of bevacizumab (Avastin) in angioid streaks Acta Ophthalmol Scand 2006 84 835 836 10.1111/j.1600-0420.2006.00762.x 17083556

[B17] Derriman L Marshall J Moorman C Downes SM The use of intravitreal bevacizumab to treat choroidal neovascular membranes (CNVMs) Retina 2008 28 910 author reply 910–911 18536613 10.1097/IAE.0b013e31816d81d0

[B18] Mimoun G Tilleul J Leys A Coscas G Soubrane G Souied EH Intravitreal ranibizumab for choroidal neovascularization in angioid streaks Am J Ophthalmol 2010 150 692 700 e691 10.1016/j.ajo.2010.06.004 20719301

[B19] Kang S Roh YJ Intravitreal ranibizumab for choroidal neovascularisation secondary to angioid streaks Eye (Lond) 2009 23 1750 1751 10.1038/eye.2009.158 19590525

[B20] Shah M Amoaku WM Intravitreal ranibizumab for the treatment of choroidal neovascularisation secondary to angioid streaks Eye (Lond) 2012 26 1194 1198 10.1038/eye.2012.116 22722486 PMC3443824

[B21] Schaal S Kaplan HJ Tezel TH Is there tachyphylaxis to intravitreal anti-vascular endothelial growth factor pharmacotherapy in age-related macular degeneration? Ophthalmology 2008 115 2199 2205 10.1016/j.ophtha.2008.07.007 18930553

[B22] Keane PA Liakopoulos S Ongchin SC Heussen FM Msutta S Chang KT Walsh AC Sadda SR Quantitative subanalysis of optical coherence tomography after treatment with ranibizumab for neovascular age-related macular degeneration Invest Ophthalmol Vis Sci 2008 49 3115 3120 10.1167/iovs.08-1689 18408176 PMC2673192

[B23] Gasperini JL Fawzi AA Khondkaryan A Lam L Chong LP Eliott D Walsh AC Hwang J Sadda SR Bevacizumab and ranibizumab tachyphylaxis in the treatment of choroidal neovascularisation Br J Ophthalmol 2012 96 14 20 10.1136/bjo.2011.204685 21791509

[B24] Sugita I Yoneda M Iwaki M Zako M Comparative Analysis of Hyaluronan’s Affinity for Antivascular Endothelial Growth Factor Agents Ophthalmic Res 2012 49 43 48 23128274 10.1159/000342975

[B25] Christoforidis JB Williams MM Wang J Jiang A Pratt C Abdel-Rasoul M Hinkle GH Knopp MV Anatomic and pharmacokinetic properties of intravitreal bevacizumab and ranibizumab after vitrectomy and lensectomy Retina 2013 33 946 952 10.1097/IAE.0b013e3182753b12 23407351 PMC4086838

[B26] Prabhu VV Morris RJ Shah PK Narendran V Combination treatment of low fluence photodynamic therapy and intravitreal ranibizumab for choroidal neovascular membrane secondary to angioid streaks in Paget’s disease - 12 month results Indian J Ophthalmol 2011 59 306 308 10.4103/0301-4738.82000 21666317 PMC3129757

[B27] Artunay O Yuzbasioglu E Rasier R Sengul A Senel A Bahcecioglu H Combination treatment with intravitreal injection of ranibizumab and reduced fluence photodynamic therapy for choroidal neovascularization secondary to angioid streaks: preliminary clinical results of 12-month follow-up Retina 2011 31 1279 1286 10.1097/IAE.0b013e318205b228 21394063

